# MAPKs signaling is obligatory for male reproductive function in a development-specific manner

**DOI:** 10.3389/frph.2024.1330161

**Published:** 2024-02-09

**Authors:** Lokesh Kumar, Subhash Solanki, Ashish Jain, Michael Botts, Rahul Gupta, Sandeep Rajput, Elon Roti Roti

**Affiliations:** ^1^ Genus Breeding India Pvt Ltd., Pune, India; ^2^ GenusPlc, ABS Global, Windsor, WI, United States; ^3^ Department of Microbiology, Smt. CHM College, University of Mumbai, Ulhasnagar, India

**Keywords:** MAPK signaling, spermatogenesis, blood testis barrier, apoptosis, low-fertile bulls, oxidative phosphorylation

## Abstract

Mitogen-activated protein kinases (MAPKs) represent widely expressed and evolutionarily conserved proteins crucial for governing signaling pathways and playing essential roles in mammalian male reproductive processes. These proteins facilitate the transmission of signals through phosphorylation cascades, regulating diverse intracellular functions encompassing germ cell development in testis, physiological maturation of spermatozoa within the epididymis, and motility regulation at ejaculation in the female reproductive tract. The conservation of these mechanisms appears prevalent across species, including humans, mice, and, to a limited extent, livestock species such as bovines. In Sertoli cells (SCs), MAPK signaling not only regulates the proliferation of immature SCs but also determines the appropriate number of SCs in the testes at puberty, thereby maintaining male fertility by ensuring the capacity for sperm cell production. In germ cells, MAPKs play a crucial role in dynamically regulating testicular cell-cell junctions, supporting germ cell proliferation and differentiation. Throughout spermatogenesis, MAPK signaling ensures the appropriate Sertoli-to-germ cell ratio by regulating apoptosis, controlling the metabolism of developing germ cells, and facilitating the maturation of spermatozoa within the cauda epididymis. During ejaculation in the female reproductive tract, MAPKs regulate two pivotal events—capacitation and the acrosome reaction essential for maintaining the fertility potential of sperm cells. Any disruptions in MAPK pathway signaling possibly may disturb the testicular microenvironment homeostasis, sperm physiology in the male body before ejaculation and in the female reproductive tract during fertilization, ultimately compromising male fertility. Despite decades of research, the physiological function of MAPK pathways in male reproductive health remains inadequately understood. The current review attempts to combine recent findings to elucidate the impact of MAPK signaling on male fertility and proposes future directions to enhance our understanding of male reproductive functions.

## 1 Introduction

Infertility & subfertility are growing challenges for livestock and humans alike; specific phenotypes and underlying mechanisms are both complex and diverse. Male-factor infertility has been attributed to deficits in both sperm production and function in humans (1). In livestock, including bovine, sub-fertility is typically attributed to deficits in traditional sperm motility and morphology parameters (2). The broad application of genetic technologies and high throughput screening platforms have started to elucidate some of the molecular regulators of male reproductive function, including members of the MAPK signaling pathways, which can lead to subfertility by disrupting any number of steps in spermatogenesis. Spermatogenesis is a tightly regulated process by which the testicular germinal epithelium produces spermatozoa (3). Spermatogenesis requires exquisite coordination of steroid hormone signals from the hypothalamic axis and paracrine signals in the testes, the dynamic interactions Sertoli cells (SCs) maintain with the testicular blood supply and spermatogonia progenitor cells, and the intrinsic spermatozoa maturation processes (4). Cell signaling mechanisms underlying each of these critical functions are regulated by mitogen-activated protein kinases (MAPKs). While the etiology of male infertility is multifactorial, the MAPK signaling pathways are indispensable, and disruption of these pathways can cause mechanistically distinct infertility phenotypes, depending upon the affected cell type and developmental stage (5–7). Deeper characterization of MAPK signaling mechanisms in male reproduction may provide targets to improve intractable subfertility and present an attractive option to develop male contraceptives. This review will summarize the role of MAPK signaling in the testes, highlight the gaps in current knowledge, and illustrate potential opportunities to target MAPK both for infertility and contraceptive therapeutics strategies.

### 1.1 MAPKs overview

Mitogen-activated protein kinases are ubiquitously expressed and evolutionarily conserved serine/threonine kinases that transmit signals through a series of phosphorylation cascades to regulate a wide variety of cellular functions. There are three different and sequentially functioning MAP kinases that function in the following order: activated MAPKKKs phosphorylate MAPKKs, which in turn activate MAPKs via dual phosphorylation (8, 9). The physiological outcomes of this signaling cascade depend on cell type and intracellular protein expression. Currently, 12 MAPKs, 7 MAPKKs, and 14 MAPKKKs have been identified in mammalian cells (Table 1), among them extracellular signal-regulated kinase (ERK1/2), p38 and c-Jun N-terminal protein kinase (JNK) are the most extensively studied mammalian MAPKs (8, 9). This diversity of kinases allows cells to finely tune specific signaling cascades.

**Table 1:** M**A**PKs.

MAPKKKs	MAPKKs	MAPKs
MEKK1, MEKK2, MEKK3 MEKK4, Tpl-2, ASK, Raf-1, Raf-A, Raf-B, TAK-1, MUK, Mos, SPRK, MST	MEK5, MKK1(MEK1), MKK2 (MEK2), MKK3, MKK4, MKK6, MKK7	p38a, p38b, p38d, p38g, ERK1, ERK2, ERK3, ERK4,ERK5 JNK1, JNK2 JNK3

**Figure 1:**
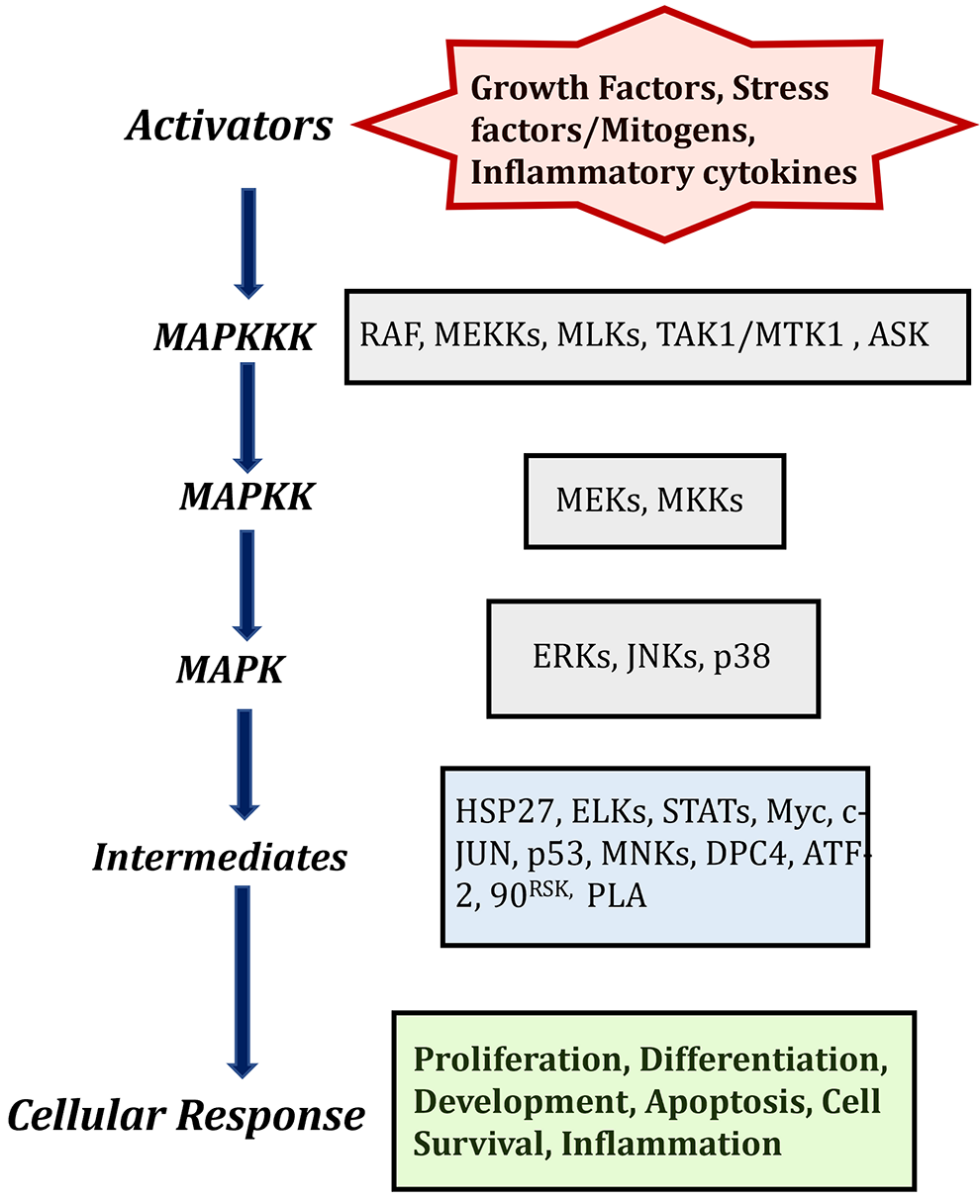
MAPK cascades in mammalian cells and description of activators and its cellular responses. Created with BioRender.com (Accessed October 2023).

The MAPK signaling pathway can be triggered by a variety of extracellular signaling molecules, including proinflammatory cytokines, mitogens, hormones, and growth factors. These signaling molecules bind to and activate their cognate receptor tyrosine kinase (RTK) at the cell surface (10, 11). Unbound RTKs reside in the cell membrane as inactive monomeric subunits. Ligand binding induces dimerization, activating the receptor and triggering conformational changes of the intracellular tyrosine kinase domain (Figure 1). The functional cellular response to MAPK signaling is dependent upon the upstream trigger, cognate receptor, and specific MAPKKKs, MAPKKs, and MAPKs that are activated the downstream intracellular targets.

## 2 MAPK signaling regulates Sertoli cell proliferation

**Figure 2:**
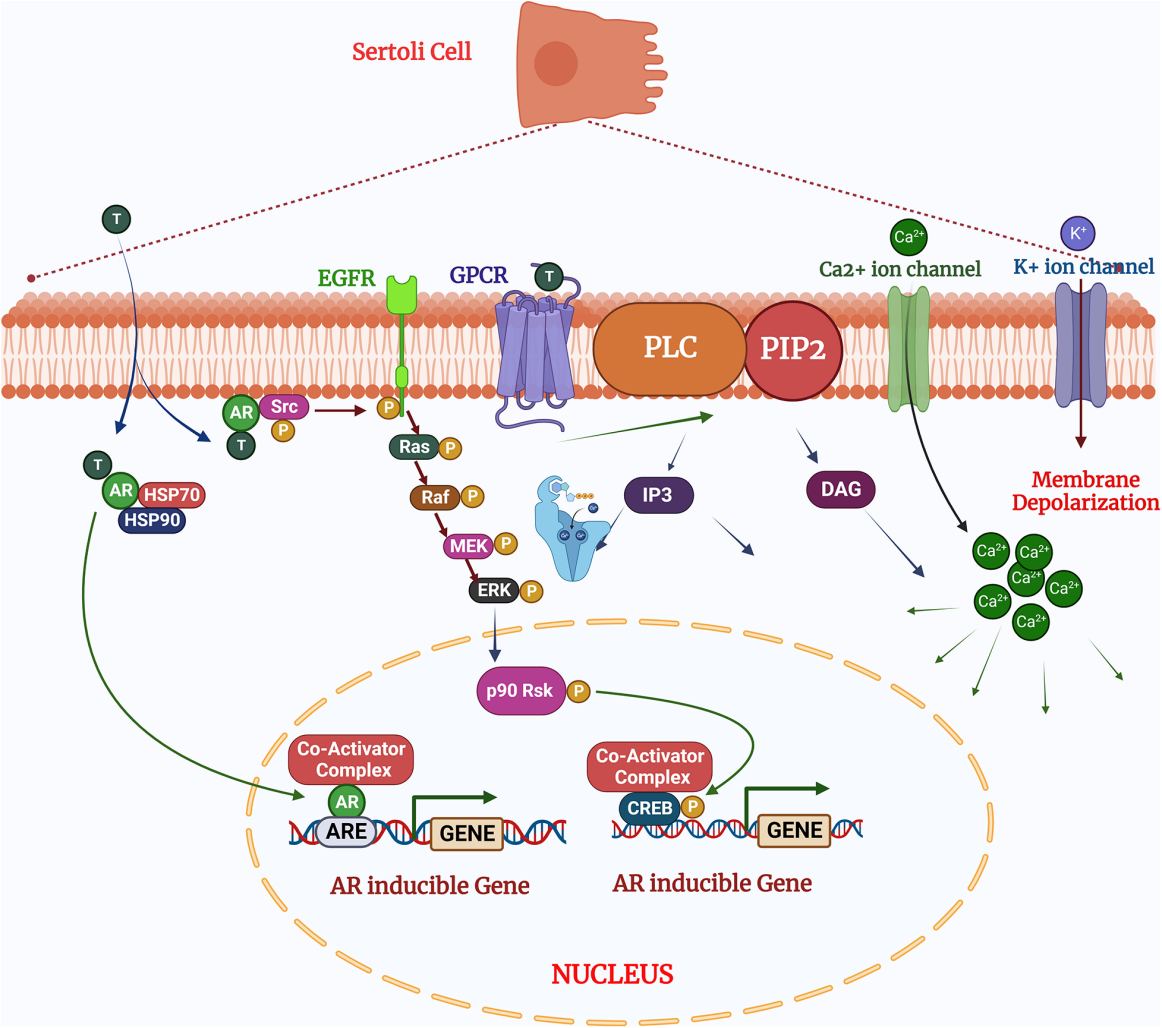
Testosterone regulates the sertoli cell function via two pathways: (1) the classical signaling pathway: testosterone diffuses by the plasma membrane and binds with the androgen receptors undergo conformational changes for activation which allowing to release HSPs (heat shock proteins) presents in the cytoplasm. After activation of cascades the androgen receptors translocate to the nucleus and binds to the specific DNA sequences androgen response elements (AREs;) and facilitates the recruitment of co-activator or co-repressor proteins which could alter the cellular functions (2). The non-classical pathways: Testosterone interacts with GPCR (G coupled G-protein coupled receptor) in the plasma membrane and activates the PLC (Phospholipase C) to cleave PIP into IP. (3) In the non-classical pathways: Testosterone binds and interact the with androgen receptors to activates Src, which activates the EGF receptors and MAPK cascade via Ras and sequentially phosphorylates and activates the RAF, MEK and ERK, resulted phosphorylates CREB on ser 133 and regulates the gene expression. Created with BioRender.com (Accessed October 2023).

The quantity and quality of testicular germ cells are determined by the number of SCs in the seminiferous tubules. SCs therefore help to maintain male fertility via defined mechanisms, transition from fetal to neonatal and pubertal stages. The immature Sertoli cells are only capable to proliferation prior to the pubertal transition and cannot undergo differentiation. The proliferation of immature SCs regulated by various signaling pathways, including MAPKs. These signaling pathways regulated upstream by the synergistic effect of FSH (follicle stimulating hormone), testosterone, and other growth factors (6). The impact of this signaling on SCs proliferation is specific to the developmental stage of the testes, which interestingly determines whether FSH either activates or inhibits the MAPK pathway. In the postnatal testis, proliferation and differentiation of SCs are mainly governed by the synergistic effect of FSH and testosterone (12). Testosterone is present at low level until the onset of puberty and diffuses into the seminiferous tubules, when LH (Luteinizing hormone) binds to the surface of Leydig cells (5, 13). FSH therefore plays more prominent role in Sertoli cell proliferation compared to testosterone, a finding which demonstrated that FSH withdrawal followed by administration of high dose testosterone, reduced the Sertoli cell mitotic index. The mitotic index was concomitantly restored after injecting FSH (14). FSH and testosterone induce ERK1/2 phosphorylation following dual coupling of FSH receptors to Gs and Gi in a heterotrimeric PKA complex. This signaling complex promotes proliferation by enhancing cyclin D1 expression leading to cell cycle progression and promoting cell growth, or size expansion (15, 16) (Figure 2). In contrast, p38 MAPK is negatively regulated by the testosterone, hence upregulation of p38 inhibits Sertoli cell maturation and reduces fertility (17, 18). This study demonstrates that the outcome of MAPK signaling is dependent upon the developmental stage of tissue, despite an identical upstream trigger and indicates MAPK signaling is critical for both neonatal Sertoli cell proliferation and germ cell-sustaining function at the adult stage.

IL-1*α* also drives the proliferation of immature SCs by activating p38 MAPK and JNK/SAPK in a manner independent of, but synergistic with FSH (15, 18). IL-1*α* does not induce ERK phosphorylation or downstream signaling in rat SCs, in contrast to FSH (15, 19). A p38 MAPK inhibitor attenuated, but did not completely block, in SCs from 8 to 9 day old rats (15, 19). p38 MAPK signaling is therefore at least partly responsible for regulating IL-1 *α*-induced proliferation, and this ERK-independent mechanism likely allows for the synergy between IL-1*α* and FSH to promote Sertoli cell mitosis**.** Based on these findings, MAPK regulation of Sertoli cell proliferation is expected to help determine the number of SCs in the testes at puberty and therefore the capacity for sperm cell production and male fertility. The use of these two independent mitogenic mechanisms likely provides exquisite tuning of Sertoli cell numbers during testicular development.

## 3 MAPKs integrate signals to dynamically regulate testicular cell-cell junctions

Spermatogenesis includes the physical migration of the germ cells from their origin in the germinal epithelium, between connected SCs, and arrival in the seminiferous tubules as spermatozoa. This physical migration requires exquisitely dynamic Sertoli-germ cell adhesion junction formation, maintenance, and dissolution as the germ cells migrate between, and interact with, SCs. Sertoli-Sertoli cell (tight) junctions must also be dynamically regulated, uncoupling to allow germ cells to migrate between the SCs, and then immediately reforming to maintain the blood-testes barrier and form interactions with the next developing germ cell (20). Mechanisms regulating junction formation and dynamics have been extensively studied (21–23); the current review will focus specifically on the role of MAPK proteins.

The evidence demonstrating MAPK signaling within the SCs is obligatory for normal cell junction dynamics is quite clear. Focusing first on the blood-testes barrier formed by the Sertoli-Sertoli tight junctions, data from rodent models demonstrate MAPK alternately promotes or inhibits tight junction stability, depending on upstream signaling. The Junction adhesion molecule B (JAM-B) is one of the required tight junction proteins, and it is the JAM-B transcript which distinct MAPK pathways either increase or decrease expression (24, 25). Positively regulating JAM-B, interluekin-1*α* activates the p38 MAPK pathway to increase JAM-B transcription, whereas in contrast, TGF-β3 activates JNK and ERK MAPK pathways to destabilize JAM-B transcript and reduce expression levels in mouse SCs (26, 27). Demonstrating this specificity for individual MAPKs, p38 inhibitors blocked interleukin-1*α*-induced JAM-B transcription and concomitant protein expression, where MAPK and ERK inhibitors has no effect on the interleukin-1*α* response (26). ERK1/2 and p54 JNK inhibitors did, however, prevent TGF-β3-induced degradation of JAM-B mRNA (27). Though JAM-B is only one of many tight junction proteins, and redundant mechanisms could exist to maintain tight junction integrity, the requirement for p38 MAPK was clearly illustrated in a subsequent Sertoli cell study (28). In cultured rat SCs, a specific p38 inhibitor prevented TGF-β3-induced loss of transepithelial electrical resistance, demonstrating p38 activity mediates the dynamic tight response to TGF-β. A MEK/ERK1/2 inhibitor had no effect on electrical resistance following TGF-β3 stimulation, however, despite the fact that the mouse study demonstrated that ERK1/2 does regulate JAM-B mRNA expression (29). This may suggest species-specific tuning of these functional pathways or indicate that p38 regulates protein(s) in addition to JAM-B that maintains tight junction electrical properties (29). Studies to date have defined MAPK mechanisms regulating testicular cell junctions only from the Sertoli cell side (29). While more challenging, and requiring an appropriate co-culture system, work to delineate whether MAPKs also regulate cell junctions from the germ cell side. This exploration is essential to attain a comprehensive understanding of the system, that could be targeted modulation of this pathway for either fertility enhancement or developing male contraceptives.

## 4 MAPK signaling coordinates testicular germ cells in a development-specific manner

Though the germ cell side of the tight junction mechanisms is not well understood, significant work has been done to characterize the role of MAPKs throughout the development process that is spermatogenesis. As the germ cells move through each stage to ultimately become spermatozoa, so too does the MAPK regulation change to drive the development continuum.

### 4.1 Germ cell proliferation and differentiation

To continuously produce large numbers of sperm cells, the testicular germ cells must consistently proliferate and differentiate; both of these processes are MAPK-dependent. In mice, glial cell-derived neurotropic factor (GDNF) stimulates both self-renewal to maintain multipotent stem cells and proliferation to maintain the spermatocyte population, while blocking differentiation of spermatogonia stem cells (30). GDNF activates the MEK pathway, increasing reactive oxygen species, and ultimately stimulating spermatogonia stem cell self-renewal via p38 and JNK signaling cascades (31). At the same time, mouse cells overexpressing GDNF are unable to respond to differentiation signals (32). These two mechanisms combine to accumulate undifferentiated spermatogonia stem cells in the testes, a mechanism which appears conserved across species.

For example, a study in bovine demonstrates that MAPK activation promotes testicular germline stem cell proliferation. Treating gonocytes cultured from neonatal bulls with GDNF activated MAPK1/2 and increased downstream cyclin D expression, known to promote cell proliferation. Inhibiting MAPK signaling in contrast limited cell proliferation and prevented colony formation. This study importantly demonstrated that MAPK regulates the renewal potential of bovine gonocytes (33).

Further down the spermatogenesis development continuum, MAPK kinase phosphorylation maintains chromatin in a highly condensed stage to avoid further DNA duplication in between consecutive meiotic divisions, as demonstrated for both mice and humans (34). This step is key to reducing the spermatogonia cell genome from diploid to haploid, and therefore necessary for sperm cell function and fertility. During late prophase I, phosphorylated ERK1 translocates to the nucleus and activates ribosomal protein S6 kinase-2 (RSK; also known as p90rsk or MAPK-activated protein kinase-1, MAPKAP-K1), forming a complex. That activated ERK1– Rsk2-p90 complex then binds to chromatin and activates cell-cycle regulator like NIMA-related protein kinase 2 (Nek-2) which in turn phosphorylates non-histone protein regulator high mobility group protein (HMGA2) (35, 36). Phosphorylated HMGA2 is released from the chromatin, a key step which allows binding of factors that trigger chromosome condensation, and thus ensures proper genomic reduction in spermatogonia cells (35, 36).

### 4.2 Apoptosis

About ∼75% of the developing germ cells normally undergo apoptosis in humans and rodents, while ∼63% do so in the buffalo testis, to maintain the appropriate Sertoli: germ cell ratio during spermatogenesis (7, 37, 38). Apoptosis is regulated by several intrinsic and extrinsic factors in a cell-specific manner during the maturation of germ cells. Any disturbance in apoptosis of developing germ cells leads to alterations in sperm production that could be one of the major causes of male infertility. Previous studies reported that heat and other inflammatory stress negatively impact testicular function, including sperm cell numbers, morphology, and physiology. Heat-stress is therefore a key factor driving seasonal breeding in many species (39–41). Low semen output during heat stress months is at least in part attributed to the induction of apoptosis in testicular germ cells (40, 42). More specifically, heat stress induces mitochondrial-dependent apoptosis in male germ cells. This is considered the “intrinsic apoptotic pathway”, and is driven by the associated signaling cascade involving cytochrome-*c* release, cleavage of PARP (poly ADP ribose polymerase), BAX translocation and activation of the caspases (43–45). This contrasts extrinsic apoptotic pathway which involves tumor necrosis factor-α (TNF-α)-driven cytokine signaling. Work combining rat and human models demonstrated MAPK activates the mitochondrial apoptotic pathway via inducible nitric oxide synthase (NOS). Using an *in vivo* GnRH and testosterone deprivation model to induce mitochondrial-dependent apoptosis and mimic the heat stress response, researchers demonstrated that p38 MAPK is activated following hormone deprivation and iNOS expression was induced in a similar time frame in rat testes. Confirming p38-MAPK directly regulates testicular apoptosis, validated using the p38-MAPK inhibitor SB202190 significantly reduced NOS activation and DNA fragmentation (an apoptotic reporter) in the human seminiferous tubules cultured in the absence of serum to induce apoptosis (46)**.**

It may be attractive to hypothesize that influencing the MAPK pathway could improve sperm cell numbers during summer heat stress, but if effective, preventing apoptosis is likely to lead to an accumulation of stressed cells which may or may not be fertilization-competent. Alternatively, the given availability of clinically applicable MAPK effectors could be considered as contraceptives. To be clinically relevant as contraceptives, these compounds would need to show consistent efficacy in eliminating sperm cell production at tolerated doses and demonstrate reversibility. In addition, localized delivery may be required to prevent undesired systemic effects given the ubiquitous nature of MAPK signaling in all cell types.

### 4.3 Metabolism

**Figure 3:**
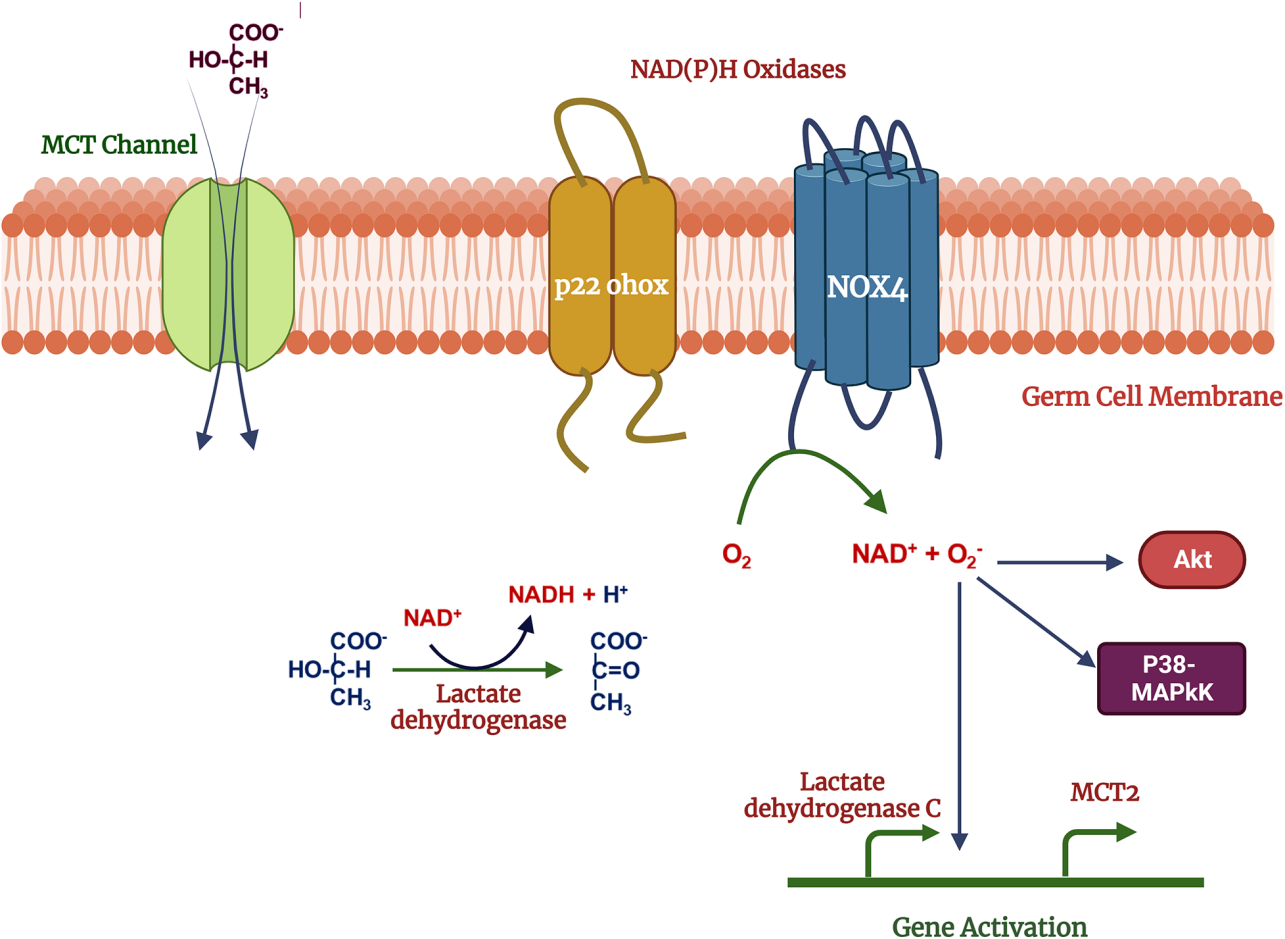
Mechanism of lactate effects on male germ cell function: lactate used as metabolic substrate by germ cells and oxidized into pyruvate which increase NADH, acts as a substrate for NOX4. Reactive oxygen species produced in the NOX4 activity, may act as second messengers activates the p38MAPK, Akt and other MAPK signal transduction pathways regulates the gene expression. Created with BioRender.com (Accessed October 2023).

Germ cells switch their metabolic profile throughout the developmental stages in the testis (47–49). Lactate provided by SCs is the primary energy substrate produced from blood glucose, the uptake of which is regulated by specific glucose transporters (GLUTs) (50). Lactate is exported from SCs by monocarboxylate transporters (MCTs) and delivered to germ cells. During the metabolic transition, a change in the cellular redox state may result in a change in intracellular ROS levels (51, 52). ROS may in turn act as signaling molecules which are involved in the activation of several signal transduction pathways such as PI3K/Akt, p38-MAPK and Erk1/2 (53, 54). Germ cells demonstrate treatment with 500 mM H_2_O_2_ and 10 mM lactate activate signal transduction cascades including PI3K/Akt, p38-MAPK and Erk1/2 in (54). Lactate is oxidized to form pyruvate, increasing NADH levels, and providing a substrate for NOX4, then activating the ROS production. ROS may act as second messengers regulating Akt and p38-MAPK signal transduction pathways and expression of MCT2 (Monocarboxylate transporter 2) and LDH C genes (Lactate dehydrogenase) (54) (Figure 3). Na ^+ ^-K^+^ ATPase (NKA) is crucial to maintaining energy hemostasis and acts as ROS-mediated signal transducer that activates the MAPK pathway, and intracellular calcium (55–57). NKA is a widely distributed transmembrane protein, *α*4 isoform (ATP1A4) exclusively present on the germ cells and sperm surface. It has been reported that NKA expression is downregulated in human asthenzoospermia compared to normozoospermia patients may lead to defects in MAPK pathways (58). In bovines, NKA may also be an important parameter of bull's sperm-fertilizing potential (59).

## 5 MAPK signaling supports spermatozoa quiescence in the cauda epididymis

Immature spermatozoa are released in the proximal part of epididymis for maturation where they pass from caput to cauda epididymis. The caudal epididymis provides an environment where fully mature spermatozoa stay in a quiescent state to conserve energy and survive until ejaculation into the female reproductive tract. Interestingly, about 90% of water is absorbed during epididymis passage to support storing a large number of sperm in that quiescent state (60, 61). An early study focused on defining the mechanisms by which spermatozoa transition from the quiescent to motile state demonstrated active p38 MAPK was higher in quiescent rat spermatozoa than motile cells, where in contrast, pERK1/2 MAPK was higher in motile cells (62). Building on these findings, Devi et al. found phosphorylated levels of p38 MAPK, Akt, mTOR, and p70S6K were greater in quiescent rat sperm compared to motile spermatozoa from the caudal epididymis (63). Activating each of these kinases in motile sperm decreased the percentage of motile cells, while inhibiting these same kinases increased the percentage of motile sperm (63). The study furthermore found that p70S6K was downstream of p38, Akt, and mTOR, suggesting that p38 acts as one of the master kinases regulating the transition from quiescent to motile sperm. Taken together, these studies suggest that ERK1/2 and p38 MAPK regulate the quiescent to motile cell transition in an opposing fashion.

## 6 MAPK signaling regulates ejaculated sperm cell function

MAPK signaling plays an important function to regulates the spermatozoa motility attainment, capacitation, and acrosome reaction that are critical events to attain the fertilization potential and establishing the successful pregnancy.

### 6.1 Sperm motility

Sperm cells must maintain a progressively motile phenotype and the ability to respond to hyperactivating signals in the female tract in order to reach the oocyte in the oviduct. Ejaculated human spermatozoa express ERK1/2, and phosphorylation of this MAPK is increased in capacitated sperm treated with either PMA or vanadate peroxide (64). These authors observed no phospho-ERK1/2 response to progesterone stimulation in their capacitated sperm cell model, where a previous publication had demonstrated opposing results (65). The p38 phosphorylation was not induced by any of the activators, demonstrating specificity of the ERK1/2 response (64). Consistent with these results a MEK1-specific inhibitor (MEK1 mediates ERK1/2 signaling) attenuated the number of motile cells in the capacitated sperm cells, while a p38 inhibitor dramatically increased the percentage of motile cells (64). These studies suggested that the reciprocal regulation of sperm cell motility by ERK1/2 and p38 MAPK is probably conserved across species. In bovines, a reduction in MAPK signaling is correlated with bull fertility (66). It has been reported that genes associated with oxidative phosphorylation of proteins involve in the MAPK signaling of sperm function are significantly down regulated in low fertile bulls (66).

### 6.2 Capacitation

Capacitation occurs as the spermatozoa transit the female reproductive tract and is required for sperm to be capable of the acrosome reaction and fertilizing the oocyte. Capacitation is characterized by biochemical, physiological and membrane rearrangement prior to the acrosome reaction including, an efflux of cholesterol leading to an increased in membrane fluidity, changes in protein kinases activity, increases permeability for bicarbonate and calcium ions, and intracellular pH (67).

In bovine, endogenous cannabinoid receptors 1/2 (CB1/2; G-protein couple receptors) present in spermatozoa are involved in capacitation and the interplay with progesterone in the female reproductive tract. The resultant acrosome reaction is activated by several pathways, ion channels and cAMP followed by downstream activation of MAPK and IP3 signaling cascade (68).

Clearly demonstrating the multifaceted nature of progesterone-induced capacitation and the acrosome reaction, work by Mahajan et al. (68) in bovine demonstrated that complete block of P4 capacitation required simultaneous block of CB1/2, TRPV1, and CatSper channels, and possibly IP3R, where blocking only one receptor type was insufficient to prevent capacitation based on evaluating classic hallmarks of phosphorylation, CTC, motility, and head staining patterns (68). Blocking MAPK signaling with PD184352 (a MEK inhibitor) in addition to the CB1/2 completely eliminated all detectable B pattern sperm cells following P4 stimulation. Blocking only CB1/2 allowed P4 to double the percentage of B-pattern spermatozoa compared to basal control levels, and similar results were obtained using the sAC inhibitor, KH7, the PKA inhibitor H-89, and the IP3R inhibitor LY294002. Data suggest that each of these intracellular signaling molecules is obligatory for the acrosome reaction. More specifically, however, CB1 and CB2 appear to trigger an increase in cAMP, which in turn activates MAPK signaling and is required to trigger the acrosome reaction. Each of the receptors therefore plays a role in at least part of the acrosome signal because blocking just one is insufficient to prevent the acrosome reaction. Blocking one receptor in combination with intracellular transducers does, however prevent the acrosome reaction, suggesting integration or redundancy in upstream signaling.

### 6.3 Acrosome reaction

The acrosome reaction is an exocytic process that releases hydrolyzing enzymes required for the sperm to invade the zona pellucida (ZP) and fertilize the oocyte (69). The physiological events underlying the acrosome reaction are characterized by the influx of calcium and subsequent protein phosphorylation (69). The phosphorylation events are regulated by MAPKs, with crosstalk to other signaling pathways including protein kinase A (PKA), protein kinase C (PKC) and Protein Tyrosine Kinase (70). ZP can bind to plasma membrane receptors on the sperm cell, including G-protein coupled receptors and tyrosine kinase receptors. Binding then activates phospholipase-C (71) which increases cAMP via adenylate cyclase and activates protein kinase A. The protein kinase A activates the voltage-dependent Ca^2+^ channel in the acrosomal membrane and causes Ca^2+^ efflux from the acrosome into the cytosol (72). Ca^2 + ^activates the Ras-Raf-ERK cascade via G-protein coupled receptor/PLC/diacylglycerol/PKC signaling in conjunction with progesterone and the bicarbonate/cAMP/Ca^2+^ pathway (73). This cAMP activates PKA, which subsequently activates Hsp90 and makes a complex with co-chaperone Cdc37 to stabilize and phosphorylate ERK1/2. The culmination of this signaling cascade promotes hyperactivation and the acrosome reaction. In contrast, the Hsp-Cdc37-ERK1/2 complex maintains p38 in an un-phosphorylated state which negatively regulates the acrosome reaction. This mechanism was elucidated using a specific ERK1/2 and Hsp90 inhibitor, which promoted the dissociation of this complex, resulting in activation of the p38 through its autophosphorylation. This ERK1/2-mediated pathway inhibits the acrosome reaction and capacitation (71, 74). This suggests that MAPKs are required for this fertilization-obligatory event; irreversible MAPK inhibitors could therefore be potential contraceptives if they specifically targeted sperm cells without affecting cells in the female tract. Further research is needed, however, to investigate the potential function of MAPK signaling in the rearrangement of cytoskeletal components during capacitation and the acrosome response.

## 7 Conclusions

Previous research has shown that MAPK activators and inhibitors can be used to treat a variety of human illnesses, including infertility and other reproductive abnormalities in both men and women using assisted reproductive procedures. In this study, we focused on the role of the MAPK signaling cascade in germ cell maturation in the testis and the generation of completely functioning mature sperm. We have provided information on the potential use of MAPK modulators and MAPK-interacting sperm-specific proteins as tools for the development of non-hormonal contraception and the management of male reproductive diseases (Table 2). This might also aid in the development of novel and more effective therapy techniques for male infertility and other associated reproductive problems. Changes in molecular mechanisms caused by stress-activated kinases like p38 and JNK, as well as their downstream effector pathways, could be utilized as markers for infertility in mammals including dairy animals. Conclusively, MAPK kinases could be a potential target for therapeutic strategies to manage male reproductive disorders. However, extensive research is needed to decipher the molecular mechanism and regulatory pathways associated with the MAPKs.

**Table 2:** MAPK inhibitors and reproductive functions.

Inhibitors	Effects on reproductive function	References
PD0325901 (MEK1/2 inhibitor)	• Inhibition of proliferation and self-renewal capacity of spermatogonia stem cells	(Hasegawa et al. 75)
U0126 (MEK1/2 inhibitor)	• Inhibition effect on BTB formation Showed protective effect during testicular transplantation. • Abolished the acrosome reaction.	(Almog et al. 64; H. Zhang et al. 76)
PD98059 (ERK 1/2 inhibitor)	• Down regulates the Apoptosis in germ cells. • Negative effect on flagellar movement of sperm. • Abolished the acrosome reaction.	(Almog et al. 64; Minutoli et al. 77)
SB203580 (p38 inhibitor)	• Down regulates the proliferation of germ lines. • Significantly increases the forward motility of sperm. • Abolished the acrosome reaction. • Downregulates the apoptosis. • Negative effect on capacitation.	(Almog et al. 64; Luna et al. 78; Morimoto et al. 31)
PD169316 (p38 inhibitor)	• Significantly increases the forward motility of sperm. • Abolished the acrosome reaction.	(Almog et al. 64; Morimoto et al. 31)
SP600125 (pJNK inhibitor)	• Down regulates the proliferation of germ lines. • A significant diminish the SSC numbers. • Downregulates the apoptosis. • Negative effect on capacitation.	(Luna et al. 2017; Morimoto et al. 31)
PD98095 (MAPK Inhibitor)	• Impaired proliferation and colony formation in bovine gonocyte culture.	(Sahare et al. 33)
PD98059 (MAPK44/42) inhibitor	• Decreased tyrosine phosphorylation of MAPK in bovine gonocyte culture.	(Sahare et al. 33)

## References

[1] Cardona Barberán A Boel A Vanden Meerschaut F Stoop D Heindryckx B. Diagnosis and treatment of male infertility-related fertilization failure. J Clin Med. (2020) 9(12):3899. 10.3390/jcm9123899 33271815 PMC7761017

[2] Kumaresan A Johannisson A Al-Essawe EM Morrell JM. Sperm viability, reactive oxygen species, and DNA fragmentation index combined can discriminate between above- and below-average fertility bulls. J Dairy Sci. (2017) 100(7):5824–36. 10.3168/jds.2016-12484 28478003

[3] Yuan Y Li L Cheng Q Diao F Zeng Q Yang X In vitro testicular organogenesis from human fetal gonads produces fertilization-competent spermatids. Cell Res. (2020) 30(3):244–55. 10.1038/s41422-020-0283-z 32086476 PMC7054335

[4] Barone B Napolitano L Abate M Cirillo L Reccia P Passaro F The role of testosterone in the elderly: what do we know? Int J Mol Sci. (2022) 23(7):3535. 10.3390/ijms23073535 35408895 PMC8998588

[5] Walker WH. Testosterone signaling and the regulation of spermatogenesis. Spermatogenesis. (2011) 1(2):116–20. 10.4161/spmg.1.2.16956 22319659 PMC3271653

[6] Shah W Khan R Shah B Khan A Dil S Liu W The molecular mechanism of sex hormones on sertoli cell development and proliferation. Front Endocrinol (Lausanne). (2021) 12:648141. 10.3389/fendo.2021.648141 34367061 PMC8344352

[7] Ni FD Hao SL Yang WX. Multiple signaling pathways in sertoli cells: recent findings in spermatogenesis. Cell Death Dis. (2019) 10(8):541. 10.1038/s41419-019-1782-z 31316051 PMC6637205

[8] Zeyen L Seternes OM Mikkola I. Crosstalk between p38 MAPK and GR signaling. Int J Mol Sci. (2022) 23(6):3322. 10.3390/ijms23063322 35328742 PMC8953609

[9] Guida E Tassinari V Colopi A Todaro F Cesarini V Jannini B MAPK Activation drives male and female mouse teratocarcinomas from late primordial germ cells. J Cell Sci. (2022) 135(8):jcs259375. 10.1242/jcs.259375 35297490

[10] Lemmon MA Schlessinger J. Cell signaling by receptor tyrosine kinases. Cell. (2010) 141(7):1117–34. 10.1016/j.cell.2010.06.011 20602996 PMC2914105

[11] Overeem AW Chang YW Spruit J Roelse CM Chuva De Sousa Lopes SM. Ligand-receptor interactions elucidate sex-specific pathways in the trajectory from primordial germ cells to gonia during human development. Front Cell Dev Biol. (2021) 9:661243. 10.3389/fcell.2021.661243 34222234 PMC8253161

[12] Pivonello R Menafra D Riccio E Garifalos F Mazzella M de Angelis C Metabolic disorders and male hypogonadotropic hypogonadism. Front Endocrinol (Lausanne). (2019) 10:345. 10.3389/fendo.2019.00345 31402895 PMC6669361

[13] Scheutz Henriksen L Holm Petersen J Skakkebæk NE Jørgensen N Virtanen HE Priskorn L Serum testosterone levels in 3-month-old boys predict their semen quality as young adults. J Clin Endocrinol Metab. (2022) 107(7):1965–75. 10.1210/clinem/dgac173 35323957 PMC9202716

[14] Meachem SJ McLachlan RI de Kretser DM Robertson DM Wreford NG. Neonatal exposure of rats to recombinant follicle stimulating hormone increases adult sertoli and spermatogenic cell numbers. Biol Reprod. (1996) 54(1):36–44. 10.1095/biolreprod54.1.36 8837998

[15] Crépieux P Marion S Martinat N Fafeur V Vern YL Kerboeuf D The ERK-dependent signalling is stage-specifically modulated by FSH, during primary Sertoli cell maturation. Oncogene. (2001) 20(34):4696–709. 10.1038/sj.onc.1204632 11498792

[16] Musnier A Heitzler D Boulo T Tesseraud S Durand G Lécureuil C Developmental regulation of p70 S6 kinase by a G protein-coupled receptor dynamically modelized in primary cells. Cell Mol Life Sci. (2009) 66(21):3487–503. 10.1007/s00018-009-0134-z 19730801 PMC11115785

[17] Gautam M Bhattacharya I Rai U Majumdar SS. Hormone induced differential transcriptome analysis of sertoli cells during postnatal maturation of rat testes. PLoS One. (2018) 13(1):e0191201. 10.1371/journal.pone.0191201 29342173 PMC5771609

[18] Luo D He Z Yu C Guan Q. Role of p38 MAPK signalling in testis development and male fertility. Oxid Med Cell Longev. (2022) 2022:6891897. 10.1155/2022/6891897 36092154 PMC9453003

[19] Petersen C Svechnikov K Fröysa B Söder O. The p38 MAPK pathway mediates interleukin-1-induced Sertoli cell proliferation. Cytokine. (2005) 32(1):51–9. 10.1016/j.cyto.2005.07.014 16181786

[20] Smith BE Braun RE. Germ cell migration across Sertoli cell tight junctions. Science. (2012) 338(6108):798–802. 10.1126/science.1219969 22997133 PMC3694388

[21] Xiao X Yang Y Mao B Cheng CY Ni Y. Emerging role for SRC family kinases in junction dynamics during spermatogenesis. Reproduction. (2019) 157(3):R85–94. 10.1530/REP-18-0440 30608903 PMC6602873

[22] Luaces JP Toro-Urrego N Otero-Losada M Capani F. What do we know about blood-testis barrier? Current understanding of its structure and physiology. Front Cell Dev Biol. (2023) 11:1114769. 10.3389/fcell.2023.1114769 37397257 PMC10307970

[23] Lv D Zhao M Ni J Liu W Ren Y Zhu D NGF Regulates sertoli cell growth and prevents LPS-induced junction protein damage via PI3K/AKT/NF*κ*B signaling. Theriogenology. (2023) 195:138–48. 10.1016/j.theriogenology.2022.10.017 36332373

[24] Stanton PG. Regulation of the blood-testis barrier. Semin Cell Dev Biol. (2016) 59:166–73. 10.1016/j.semcdb.2016.06.018 27353840

[25] Han X Zhang C Ma X Yan X Xiong B Shen W Muscarinic acetylcholine receptor M5 is involved in spermatogenesis through the modification of cell-cell junctions. Reproduction. (2021) 162(1):47–59. 10.1530/REP-21-0079 33970124 PMC8183636

[26] Wang Y Lui WY. Opposite effects of interleukin-1alpha and transforming growth factor-beta2 induce stage-specific regulation of junctional adhesion molecule-B gene in Sertoli cells. Endocrinology. (2009) 150(5):2404–12. 10.1210/en.2008-1239 19164472

[27] Zhang X Lui WY. Transforming growth factor-β3 regulates cell junction restructuring via MAPK-mediated mRNA destabilization and smad-dependent protein degradation of junctional adhesion molecule B (JAM-B). Biochim Biophys Acta. (2015) 1849(6):601–11. 10.1016/j.bbagrm.2015.03.005 25817991

[28] Zhang C Lu D Niu T Sun Z Wang Y Han X LncRNA5251 inhibits spermatogenesis via modification of cell-cell junctions. Biol Direct. (2023) 18(1):31. 10.1186/s13062-023-00381-x 37316926 PMC10268499

[29] Ma Q You X Zhu K Zhao X Yuan D Wang T Changes in the tight junctions of the testis during aging: role of the p38 MAPK/MMP9 pathway and autophagy in sertoli cells. Exp Gerontol. (2022) 161:111729. 10.1016/j.exger.2022.111729 35134475

[30] Meng X Lindahl M Hyvönen ME Parvinen M de Rooij DG Hess MW Regulation of cell fate decision of undifferentiated spermatogonia by GDNF. Science. (2000) 287(5457):1489–93. 10.1126/science.287.5457.1489 10688798

[31] Morimoto H Iwata K Ogonuki N Inoue K Atsuo O Kanatsu-Shinohara M ROS Are required for mouse spermatogonial stem cell self-renewal. Cell Stem Cell. (2013) 12(6):774–86. 10.1016/j.stem.2013.04.001 23746981

[32] Yomogida K Yagura Y Tadokoro Y Nishimune Y. Dramatic expansion of germinal stem cells by ectopically expressed human glial cell line-derived neurotrophic factor in mouse Sertoli cells. Biol Reprod. (2003) 69(4):1303–7. 10.1095/biolreprod.103.015958 12801989

[33] Sahare M Otomo A Komatsu K Minami N Yamada M Imai H. The role of signaling pathways on proliferation and self-renewal of cultured bovine primitive germ cells. Reprod Med Biol. (2015) 14(1):17–25. 10.1007/s12522-014-0189-x 29259399 PMC5715818

[34] Li MWM Mruk DD Cheng CY. Mitogen-activated protein kinases in male reproductive function. Trends Mol Med. (2009) 15(4):159–68. 10.1016/j.molmed.2009.02.002 19303360 PMC2804913

[35] Di Agostino S Rossi P Geremia R Sette C. The MAPK pathway triggers activation of Nek2 during chromosome condensation in mouse spermatocytes. Development. (2002) 129(7):1715–27. 10.1242/dev.129.7.1715 11923207

[36] Di Agostino S Fedele M Chieffi P Fusco A Rossi P Geremia R Phosphorylation of high-mobility group protein A2 by Nek2 kinase during the first meiotic division in mouse spermatocytes. Mol Biol Cell. (2004) 15(3):1224–32. 10.1091/mbc.e03-09-0638 14668482 PMC363112

[37] Huang YL Zhang PF Hou Z Fu Q Li MX Huang DL Ubiquitome analysis reveals the involvement of lysine ubiquitination in the spermatogenesis process of adult Buffalo (bubalus bubalis) testis. Biosci Rep. (2020) 40(6):BSR20193537. 10.1042/BSR20193537 32469046 PMC7298129

[38] Zhao H Ma N Chen Q You X Liu C Wang T Decline in testicular function in ageing rats: changes in the unfolded protein response and mitochondrial apoptotic pathway. Exp Gerontol. (2019) 127:110721. 10.1016/j.exger.2019.110721 31491500

[39] Yadav SK Pandey A Kumar L Devi A Kushwaha B Vishvkarma R The thermo-sensitive gene expression signatures of spermatogenesis. Reprod Biol Endocrinol. (2018) 16(1):56. 10.1186/s12958-018-0372-8 29859541 PMC5985054

[40] Zhang SX Wang DL Qi JJ Yang YW Sun H Sun BX Chlorogenic acid ameliorates the heat stress-induced impairment of porcine Sertoli cells by suppressing oxidative stress and apoptosis. Theriogenology. (2024) 214:148–56. 10.1016/j.theriogenology.2023.10.018 37875054

[41] Hansen PJ. Effects of heat stress on mammalian reproduction. Philos Trans R Soc Lond B Biol Sci. (2009) 364(1534):3341–50. 10.1098/rstb.2009.0131 19833646 PMC2781849

[42] Yin Y Hawkins KL DeWolf WC Morgentaler A. Heat stress causes testicular germ cell apoptosis in adult mice. J Androl. (1997) 18(2):159–65. 10.1002/j.1939-4640.1997.tb01896.x 9154510

[43] Chakraborty A Singh V Singh K Rajender S. Excess iodine impairs spermatogenesis by inducing oxidative stress and perturbing the blood testis barrier. Reprod Toxicol. (2020) 96:128–40. 10.1016/j.reprotox.2020.06.012 32593569

[44] Ding X Ge B Wang M Zhou H Sang R Yu Y Inonotus obliquus polysaccharide ameliorates impaired reproductive function caused by toxoplasma gondii infection in male mice via regulating Nrf2-PI3K/AKT pathway. Int J Biol Macromol. (2020) 151:449–58. 10.1016/j.ijbiomac.2020.02.178 32084465

[45] Frasor J Barnett DH Danes JM Hess R Parlow AF Katzenellenbogen BS. Response-specific and ligand dose-dependent modulation of estrogen receptor (ER) alpha activity by ERbeta in the uterus. Endocrinology. (2003) 144(7):3159–66. 10.1210/en.2002-0143 12810572

[46] Vera Y Erkkilä K Wang C Nunez C Kyttänen S Lue Y Involvement of p38 mitogen-activated protein kinase and inducible nitric oxide synthase in apoptotic signaling of murine and human male germ cells after hormone deprivation. Mol Endocrinol. (2006) 20(7):1597–609. 10.1210/me.2005-0395 16469770

[47] Rato L Alves MG Socorro S Duarte AI Cavaco JE Oliveira PF. Metabolic regulation is important for spermatogenesis. Nat Rev Urol. (2012) 9(6):330–8. 10.1038/nrurol.2012.77 22549313

[48] Boussouar F Benahmed M. Lactate and energy metabolism in male germ cells. Trends Endocrinol Metab. (2004) 15(7):345–50. 10.1016/j.tem.2004.07.003 15350607

[49] Voigt AL Kondro DA Powell D Valli-Pulaski H Ungrin M Stukenborg JB Unique metabolic phenotype and its transition during maturation of juvenile male germ cells. FASEB J. (2021) 35(5):e21513. 10.1096/fj.202002799R 33811704 PMC8212869

[50] Schrade A Kyrönlahti A Akinrinade O Pihlajoki M Fischer S Rodriguez VM GATA4 Regulates blood-testis barrier function and lactate metabolism in mouse Sertoli cells. Endocrinology. (2016) 157(6):2416–31. 10.1210/en.2015-1927 26974005 PMC4891789

[51] Bassenge E Sommer O Schwemmer M Bünger R. Antioxidant pyruvate inhibits cardiac formation of reactive oxygen species through changes in redox state. Am J Physiol Heart Circ Physiol. (2000) 279(5):H2431–8. 10.1152/ajpheart.2000.279.5.H2431 11045981

[52] Brooks GA. Cell-cell and intracellular lactate shuttles. J Physiol. (2009) 587(Pt 23):5591–600. 10.1113/jphysiol.2009.178350 19805739 PMC2805372

[53] Tai P Ascoli M. Reactive oxygen species (ROS) play a critical role in the cAMP-induced activation of ras and the phosphorylation of ERK1/2 in leydig cells. Mol Endocrinol. (2011) 25(5):885–93. 10.1210/me.2010-0489 21330403 PMC3386528

[54] Galardo MN Regueira M Riera MF Pellizzari EH Cigorraga SB Meroni SB. Lactate regulates rat male germ cell function through reactive oxygen species. PLoS One. (2014) 9(1):e88024. 10.1371/journal.pone.0088024 24498241 PMC3909278

[55] Zhang J Wang X Vikash V Ye Q Wu D Liu Y ROS and ROS-mediated cellular signaling. Oxid Med Cell Longev. (2016) 2016:4350965. 10.1155/2016/4350965 26998193 PMC4779832

[56] Rajamanickam GD Kroetsch T Kastelic JP Thundathil JC. Testis-specific isoform of Na/K-ATPase (ATP1A4) regulates sperm function and fertility in dairy bulls through potential mechanisms involving reactive oxygen species, calcium and actin polymerization. Andrology. (2017) 5(4):814–23. 10.1111/andr.12377 28597551

[57] Samanta K Douglas S Parekh AB. Mitochondrial calcium uniporter MCU supports cytoplasmic Ca2^+^oscillations, store-operated Ca2^+^entry and Ca2+-dependent gene expression in response to receptor stimulation. PLoS One. (2014) 9(7):e101188. 10.1371/journal.pone.0101188 25004162 PMC4086884

[58] Tiwari S Rajamanickam G Unnikrishnan V Ojaghi M Kastelic JP Thundathil JC. Testis-specific isoform of Na+-K+ ATPase and regulation of bull fertility. Int J Mol Sci. (2022) 23(14):7936. 10.3390/ijms23147936 35887284 PMC9317330

[59] Thundathil JC Rajamanickam GD Kastelic JP. Na/K-ATPase and regulation of sperm function. Anim Reprod. (2018) 15(Suppl 1):711–20. 10.21451/1984-3143-AR2018-0024 36249829 PMC9536046

[60] Cornwall GA. New insights into epididymal biology and function. Hum Reprod Update. (2009) 15(2):213–27. 10.1093/humupd/dmn055 19136456 PMC2639084

[61] Correia S Oliveira PF Guerreiro PM Lopes G Alves MG Canário AVM Sperm parameters and epididymis function in transgenic rats overexpressing the Ca2+-binding protein regucalcin: a hidden role for Ca2^+^in sperm maturation? Mol Hum Reprod. (2013) 19(9):581–9. 10.1093/molehr/gat030 23615721

[62] Kumar L Yadav SK Kushwaha B Pandey A Sharma V Verma V Energy utilization for survival and fertilization-parsimonious quiescent sperm turn extravagant on motility activation in rat. Biol Reprod. (2016) 94(4):96. 10.1095/biolreprod.115.137752 26984998

[63] Devi A Kushwaha B Maikhuri JP Singh R Gupta G. Cell signaling in sperm midpiece ensures quiescence and survival in cauda epididymis. Reproduction. (2021) 162(5):339–51. 10.1530/REP-21-0202 34486982

[64] Almog T Lazar S Reiss N Etkovitz N Milch E Rahamim N Identification of extracellular signal-regulated kinase 1/2 and p38 MAPK as regulators of human sperm motility and acrosome reaction and as predictors of poor spermatozoan quality. J Biol Chem. (2008) 283(21):14479–89. 10.1074/jbc.M710492200 18372245

[65] Luconi M Krausz C Barni T Vannelli GB Forti G Baldi E. Progesterone stimulates p42 extracellular signal-regulated kinase (p42erk) in human spermatozoa. Mol Hum Reprod. (1998) 4(3):251–8. 10.1093/molehr/4.3.251 9570271

[66] Paul N Kumaresan A Das Gupta M Nag P Guvvala PR Kuntareddi C Transcriptomic profiling of buffalo spermatozoa reveals dysregulation of functionally relevant mRNAs in low-Fertile bulls. Front Vet Sci. (2020) 7:609518. 10.3389/fvets.2020.609518 33506000 PMC7829312

[67] Sansegundo E Tourmente M Roldan ERS. Energy metabolism and hyperactivation of spermatozoa from three mouse Species under capacitating conditions. Cells. (2022) 11(2):220. 10.3390/cells11020220 35053337 PMC8773617

[68] Mahajan A Sharma P Mishra AK Gupta S Yadav S Anand M Interplay mechanisms between progesterone and endocannabinoid receptors in regulating bull sperm capacitation and acrosome reaction. J Cell Physiol. (2022) 237(7):2888–912. 10.1002/jcp.30753 35476800

[69] Morohoshi A Miyata H Tokuhiro K Iida-Norita R Noda T Fujihara Y Testis-enriched ferlin, FER1L5, is required for Ca2+-activated acrosome reaction and male fertility. Sci Adv. (2023) 9(4):eade7607. 10.1126/sciadv.ade7607 36696506 PMC9876558

[70] Bogoyevitch MA Glennon PE Andersson MB Clerk A Lazou A Marshall CJ Endothelin-1 and fibroblast growth factors stimulate the mitogen-activated protein kinase signaling cascade in cardiac myocytes. The potential role of the cascade in the integration of two signaling pathways leading to myocyte hypertrophy. J Biol Chem. (1994) 269(2):1110–9. 10.1016/S0021-9258(17)42228-9 7507104

[71] du Plessis SS Page C Franken DR. The zona pellucida-induced acrosome reaction of human spermatozoa involves extracellular signal-regulated kinase activation. Andrologia. (2001) 33(6):337–42. 10.1046/j.1439-0272.2001.00449.x 11736793

[72] Ickowicz D Finkelstein M Breitbart H. Mechanism of sperm capacitation and the acrosome reaction: role of protein kinases. Asian J Androl. (2012) 14(6):816–21. 10.1038/aja.2012.81 23001443 PMC3720105

[73] Publicover S Harper C V Barratt C. [Ca2+]i signalling in sperm–making the most of what you’ve got. Nat Cell Biol. (2007) 9(3):235–42. 10.1038/ncb0307-235 17330112

[74] Sun P Wang Y Gao T Li K Zheng D Liu A Hsp90 modulates human sperm capacitation via the Erk1/2 and p38 MAPK signaling pathways. Reprod Biol Endocrinol. (2021) 19(1):39. 10.1186/s12958-021-00723-2 33663544 PMC7931335

[75] Hasegawa K Namekawa SH Saga Y. MEK/ERK signaling directly and indirectly contributes to the cyclical self-renewal of spermatogonial stem cells. Stem Cells. (2013) 31(11):2517–27. 10.1002/stem.1486 23897718 PMC3834200

[76] Zhang H Yin Y Wang G Liu Z Liu L Sun F. Interleukin-6 disrupts blood-testis barrier through inhibiting protein degradation or activating phosphorylated ERK in Sertoli cells. Sci Rep. (2014) 4:4260. 10.1038/srep04260 24584780 PMC3939460

[77] Minutoli L Antonuccio P Polito F Bitto A Squadrito F Di Stefano V Mitogen-activated protein kinase 3/mitogen-activated protein kinase 1 activates apoptosis during testicular ischemia-reperfusion injury in a nuclear factor-kappaB-independent manner. Eur J Pharmacol. (2009) 604(1-3):27–35. 10.1016/j.ejphar.2008.12.028 19135439

[78] Luna C Mendoza N Casao A Pérez-Pé R Cebrián-Pérez JA Muiño-Blanco T. c-Jun N-terminal kinase and p38 mitogen-activated protein kinase pathways link capacitation with apoptosis and seminal plasma proteins protect sperm by interfering with both routes^†^. Biol Reprod. (2017) 96(4):800–15. 10.1093/biolre/iox017 28379343

